# Cystitis

**Published:** 1894-12-08

**Authors:** G. Munro Smith

**Affiliations:** Senior Assistant Surgeon, Bristol Royal Infirmary


### Medical Progress and Hospital Clinics.

[*The Editor will be glad to receive offers of co-operation and contributions from members of the profession. All letters should be addressed to* THE EDITOR, THE LODGE, PORCHESTER SQUARE, LONDON, W.]

### CYSTITIS.

By G. MUNRO SMITH, L.R.C.P.Lond., M.R.C.S., Senior Assistant Surgeon, Bristol Royal Infirmary.

### 1.—ETIOLOGY.

The most carefully protected parts are not always proof against the entrance of the ubiquitous microbe. It may almost be said of the animal body that if the soil be suitable there it will assuredly grow, although the growth may not be vigorous, and the germ itself may be harmless or even salutary.

The male bladder is a good illustration of this fact. It is so well guarded by a long, closed passage (the urethra) from external influences that it is difficult to see how germs can enter except on a catheter or sound. Indeed, the prevailing opinion of late years has been that this is the only possible means of admission, and that however inflamed or diseased the mucous membrane of the bladder may be, there can be no true cystitis without germs, and no germs without the use of an unclean catheter. That this is the most frequent origin of cystitis must be admitted. The man with enlarged prostate or stricture may suffer pain and discomfort, and his urine may be unhealthy, but it is not in the vast majority of cases until he has sought relief from the catheter that the urine becomes putrid. It is not too much to say that this catheterisation, which appears at first such an unspeakable boon, is often the signing of the death-warrant. It is wonderful how recklessly these invisible foes, the microbes, are introduced in the place prepared for them by unhealthy conditions. Most surgeons know of patients who carry about with them a soft gum elastic catheter, usually doubled up in the coat pocket, and use it without any real attempt at cleansing it, smearing on a little salad oil or vaseline to facilitate its passage. The marvel is that infection does not occur more frequently and sooner than it does. That such cases do not at once get cystitis goes far to show that the absorptive power of the mucous membrane of the bladder is very slight; and the same may be said of the urethra that has been altered by formation of cicatricial tissue, although the normal or acutely inflamed urethra absorbs readily. It should also be remembered that the mere presence of germs and their products in a viscus does not necessarily produce any ill-effects. It is only when the products of their activity are retained for a considerable time or exist in a nutritious fluid that harm ensues. It has been shown again and again that free drainage from any cavity prevents damage by these organisms, even if they continue to exist and flourish. And this is an important point in the causation of cystitis, for it is precisely in those cases where the "drainage tube," *i.e.*, the urethra, gets partially blocked by a stricture or enlarged prostate that the microbes have sufficient time to decompose the urine and cause the disease.

But is it possible to get cystitis without germs? and if not, are they always introduced per urethram? The first question is rendered difficult to answer, because the term "cystitis" may be defined in various ways. If the urine is thick, the temperature raised, and pain accompanies micturition, it is sometimes concluded that the patient has cystitis. But this is erroneous. The term means something more definite than this, viz., a decomposition of urine taking place in the bladder. Unless this is borne in mind much confusion will result. For example: After the passage of a perfectly clean catheter "urethral fever" frequently ensues, and this, too, in cases where germs already swarm in the bladder. It would be ridiculous to ascribe this to the action of microbes. It is sudden and transitory, and can only be explained by some reflex nerve disturbance. Dr. Lydston ("International Medical Magazine," Vol. II., 1894) argues that the cases of general "infection" following operations on the genito-urinary organs may be accounted for by abnormal tissue metabolism resulting from "shock" brought about reflexly; and that germs have nothing to do with it. There is no doubt, moreover, that nervous influences profoundly modify various secretions, and it is possible that the urine may be rendered poisonous in much the same way as the mammary secretion may be rendered unfit for the child by a violent fit of anger in the mother. But if this change is possible, it does not explain the decomposition in the bladder. An attempt has been made to account for this as follows: Over distension from obstruction causes congestion of the mucous membrane and the free secretion of mucus, and the latter contains a ferment which is capable of decomposing urea into carbonate of ammonia; this again makes the urine alkaline or neutral, and precipitates the triple phosphates. It is doubtful if the most abundant secretion of mucus could cause this chain of events. There is no proof of it, and it is unnecessary, for the simple reason that germs are invariably present in cystitis. No urine from any case has ever been properly examined without detecting their presence; and as almost any microbe may effect this decomposition (it is not requisite to have the micrococcus ureæ) we need go no further in our search for a cause. Besides the above mentioned micrococcus ureæ and the bacterium ureæ (evidently stages in the life history of the same organism), numerous putrefactive and other germs have been found. Hallé, Krögius, Rorsing, &c., have described them. It may be noted that according to Du Mesnil ("Virchow's Archives," 1891), gonococci do not thrive in the bladder; they are destroyed by the urine, and the existence of a specific gonorrhœal cystitis is doubtful.

It is important also to notice that another germ found in the bladder is apparently identical with the bacterium coli commune; this will be referred to later. We may now pass on to the second queston. If germs are necessary for the production of this disease, are they always introduced per urethram? This has been frequently discussed, and men of large experience strenuously maintain that catheterisation is alone answerable for cystitis.

Professor Guyon, in 1892, wrote: "Spontaneous infection of the bladder, apart from disease of the urethra and obstruction to the flow of urine, does not exist," and he denies that the irritation of a stone can produce cystitis. He insists that the term "calculous cystitis" is a case of "putting the cart before the horse," and that the alkaline or neutral reaction of the urine brought about by germs produces the deposition of the calculus, not *vice versa*. "A calculus," he says ("Le Progrès Médical," Aug. 13th, 1892), "may cause excessive irritation of the bladder, with pus, frequent micturition, and so on, but it can only *prepare* the bladder for cystitis; it is the entrance of germs which is the exciting cause."

Dr. Bazy, however, has published cases of true cystitis occurring in patients with normal urethræ who had never had any instrument passed. He believes that the microbes from distant organs, *e.g.*, the lungs, enter the bladder. These organisms are (he says) eliminated by the kidneys without injury to those organs, but are arrested in the bladder if there is stricture, calculus, enlarged prostate, or any other cause of retention. This origin of cystitis from "descending infection" has been described by M. Jacobson in "Le Progrès Médical" for May 12th, 1894.

M. Emile Reymond ("Annales des Mal. des Organs Génito. Urin.," 1893, Nos. 4, 5, 10) has cited cases of the disease in which no instrument had been passed, and he has discovered the bacterium coli commune, which he thinks enters the bladder from the rectum. Other cases of cystitis apart from catheterisation have been reported.

On the whole, therefore, the second question: Do the germs which cause cystitis always enter by the urethra? may be answered in the negative. There seems sufficient recent evidence to show that they are introduced from the peritoneal cavity, by direct passage through the vesical walls, from the rectum, and from remote organs through the medium of the blood.

